# Evaluation of the factor structure of the Canine Behavioural Assessment and Research Questionnaire (C-BARQ) in European Portuguese

**DOI:** 10.1371/journal.pone.0209852

**Published:** 2018-12-27

**Authors:** Rute Canejo-Teixeira, Pedro Armelim Almiro, James A. Serpell, Luís V. Baptista, Maria M. R. E. Niza

**Affiliations:** 1 Centro de Investigação Interdisciplinar de Sanidade Animal, Faculdade de Medicina Veterinária/ULisboa, Lisbon, Portugal; 2 Centro de Investigação em Neuropsicologia e Intervenção Cognitivo-Comportamental, Laboratório de Avaliação Psicológica e Psicometria, Faculdade de Psicologia e de Ciências da Educação da Universidade de Coimbra, Coimbra, Portugal; 3 Center for the Interaction of Animals and Society, University of Pennsylvania School of Veterinary Medicine, Philadelphia, Pennsylvania, United States of America; 4 Faculdade de Ciências Sociais e Humanas, Universidade Nova de Lisboa, Lisboa, Portugal; Stanford University, UNITED STATES

## Abstract

The human-dog relationship is thought to be the oldest domestic animal partnership. These relationships are complex and can become problematic when they become dysfunctional. The most common signs of dysfunctional human-dog partnerships are behaviour problems that, when unidentified and uncorrected, can be a clear danger to both species and the public. The Canine Behavioural Assessment and Research Questionnaire (C-BARQ) is a widely implemented instrument to evaluate dog behaviour proven to be useful across various cultures. A European Portuguese 78-item version based on the 100-item C-BARQ was developed and its psychometric properties evaluated. The resulting questionnaire has a 13-factor structure accounting for 58.42% of the total variance with Cronbach’s alpha values ranging from 0.902 and 0.721, showing excellent to respectable consistency. The original factors, Dog-Directed Aggression and Dog-Directed Fear, both loaded strongly onto a joint factor renamed Dog Associated Fear/Aggression, explaining the 13-factor structure compared to the previously found 14-factor structure. In the European Portuguese C-BARQ only two items did not load onto their expected factor. Results show that the questionnaire measures universal dog behaviours that are evident to most owners. Our results suggest that the European Portuguese version of the C-BARQ can be used to characterize the behaviour of dog populations and is adequate for use in animal shelters to help match dogs with new owners and in clinical settings to identify behaviour problems in veterinary patients before they become unmanageable. The European Portuguese C-BARQ could be of vital importance in helping to resolve behavioural problems in owned dogs before they become so serious as to lead to abandonment or euthanasia, diminishing the pressure on municipal kennels and greatly improving canine welfare.

## Introduction

The human-dog relationship is thought to be the oldest domestic animal partnership [1], serving the needs of both the human and the dog in a wide variety of ways [2]. However, these relationships are complex and can become problematic for humans and dogs when they become dysfunctional. One of the most common signs of dysfunctional human-dog partnerships are behaviour problems that, when unidentified and uncorrected, can present a clear and present danger to both species. Dogs with unidentified behavioural problems tend to be the ones that bite humans and other animals [3], that are returned more frequently after adoption [4,5] and are most likely to be euthanized at the owners’ request. In fact, it has been suggested that behaviour problems represent the single most cited reason for the relinquishing and euthanasia of dogs [4,6,7]. As such, identifying behaviour problems before they become larger issues is important in guaranteeing both dog and human health and safety. Once identified, most of these problems can be corrected, helping to change dysfunctional human-dog dyads into functional ones.

To identify problem behaviours and understand their origin, the dog’s behaviour must be evaluated. In general, direct behavioural observation by trained behaviourists is the preferred form of assessing and classifying dog behaviour. Various tests have been developed to do so, mainly in the form of test batteries, ratings of individual dogs, expert ratings of breed prototypes, and observational tests [8]. These tests are often time consuming, require specific settings [9], their results may depend on the experimental conditions [10], and they may be difficult to conduct on a larger and more varied population, making generalization across populations difficult [8]. One way around these issues is by using the knowledge an owner possesses about the dog to evaluate an individual dog’s behaviour and temperament [11]. Although not specifically trained to observe canine behaviour, simply by virtue of their co-habitation, an owner may be knowledgeable about their pet’s behaviour. As such, owners may represent a reliable source of information regarding their dog’s behaviour.

One way to quantify owner knowledge is through questionnaires such as the widely-used Canine Behavioural Assessment and Research Questionnaire (C-BARQ), a 100-item instrument originally developed in the USA [12,13]. So far, the C-BARQ has been used to evaluate canine behaviour and screen for appropriate temperament in dogs in guide dog programs [14,15], to identify specific behaviours related to the dogs’ hormonal response to human contact [16], and even to classify behaviour phenotypes in morphological and genetic studies [17,18]. The psychometric properties of the C-BARQ have been studied in a variety of countries and validated for use in Mandarin [19], Japanese [20], Dutch [21], Swedish [11], Italian [22], Farsi [23], Latin American Spanish [24], and Brazilian Portuguese [25], making it a tool that has shown consistency and validity in assessing dog behaviour in a wide variety of cultures. Common canine behavioural problems in various populations may have common origins, or they may be unique to specific cultures; using the same validated instrument makes such comparisons possible [26]. By identifying behaviour problems present in a given population, it becomes possible to develop educational programs for owners which would focus on prevention of these issues. Through owner education, it should be possible to reduce problem behaviour, leading to a reduction in the relinquishment and euthanasia of dogs, as well as human-directed aggression [27,28].

In Portugal, dog ownership has gone through many changes in the past 20 to 30 years, since the revolution of 1974, when dogs started to become more common inside the home. It has only been very recently that dog training classes have been made available to the public which, along with the increased availability of pet insurance, demonstrates a gradual cultural shift in how the Portuguese view the family dog. Despite this shift, Portugal continues to have a dog abandonment problem, with official numbers from 2017 citing 24,079 dogs accepted in municipal kennels, of which 31% were euthanized (personal communication, National Authority for Animal Health, Government of Portugal). With the approval of the new Animal Welfare Act of 2016 [29], in which the euthanasia of healthy dogs under municipal care has been prohibited, it is likely that the importance of correct rehoming of relinquished dogs will become even more important. As such, having a reliable and valid tool, such as the C-BARQ, to assess and correctly classify a particular dog’s behavioural characteristics in a quick, easy, and consistent way could greatly benefit municipal kennels. The C-BARQ could also serve to help clarify the behavioural characteristics of the Portuguese dog population, thereby helping to direct public education campaigns that may contribute to more responsible dog ownership. In a clinical setting, the use of the C-BARQ could help veterinarians to clearly identify problems and, as such, better help owners when behaviour issues begin to appear.

The present study aims to establish the psychometric properties of an adapted and shortened 78-item European Portuguese version of the C-BARQ. Such a questionnaire may be useful in classifying dogs for rehoming as well as identifying possible behavioural problems in owned dogs before they become so serious as to lead to abandonment or euthanasia.

## Materials and methods

### Participants

All participants in this study were over 18 years of age and residents and/or citizens of Portugal. Each individual that participated was required to have owned at least one dog in his or her lifetime.

### Instrument

The version of the C-BARQ used in the current study was based on the 100-item version used in the study by Duffy and Serpell [13], itself an updated version of the original C-BARQ [12]. The questionnaire’s 100 items ask owners to assess their dog’s reactions in everyday situations and score them on a Likert-type 5-point scale of frequency (0 representing “never”, 4 representing “always”) and of severity (0 indicating “no sign of the behaviour” and 4 indicating “severe demonstrations of the behaviour”). The questionnaire was translated from English to Portuguese, corrected by three university professors, and back translated by a native English speaker (Canadian citizen). The questionnaire was then administered to a small test population of owners (*N* = 50) and, after frequent comments regarding the perceived excessive length, items labelled as “miscellaneous” (items 77 to 90), were removed to shorten the questionnaire. The result was a European Portuguese version of the C-BARQ containing 78 items (Table 1), maintaining the 7 sections of the original, but excluding 22 Miscellaneous items.

**Table 1:** CBARQ sections and items translated into European Portuguese.

Section 1: Training difficulty (frequency)
1. When off the leash, returns immediately when called.
2. Obeys the “sit” command immediately.
3. Obeys the “stay” command immediately.
4. Seems to attend/listen closely to everything you say or do.
5. Slow to respond to correction or punishment; ‘thick-skinned’.
6. Slow to learn new tricks or tasks.
7. Easily distracted by interesting sights, sounds or smells.
8. Will ‘fetch’ or attempt to fetch sticks, balls, or objects.
Section 2: Aggression (severity)
9. When verbally corrected or punished (scolded, shouted at, etc.) by you or a household member.
10. When approached directly by an unfamiliar adult while being walked/exercised on a leash.
11. When approached directly by an unfamiliar child while being walked/exercised on a leash.
12. Toward unfamiliar persons approaching the dog while s/he is in your car (at the gas station for example).
13. When toys, bones or other objects are taken away by a household member.
14. When bathed or groomed by a household member.
15. When an unfamiliar person approaches you or another member of your family at home.
16. When unfamiliar persons approach you or another member of your family away from your home.
17. When approached directly by a household member while s/he (the dog) is eating.
18. When mailmen or other delivery workers approach your home.
19. When his/her food is taken away by a household member.
20. When strangers walk past your home while your dog is outside or in the yard.
21. When an unfamiliar person tries to touch or pet the dog.
22. When joggers, cyclists, rollerbladers or skateboarders pass your home while your dog is outside or in the yard.
23. When approached directly by an unfamiliar male dog while being walked/exercised on a leash.
24. When approached directly by an unfamiliar female dog while being walked/exercised on a leash.
25. When stared at directly by a member of the household.
26. Toward unfamiliar dogs visiting your home.
27. Toward cats, squirrels or other small animals entering your yard.
28. Toward unfamiliar persons visiting your home.
29. When barked, growled, or lunged at by another (unfamiliar) dog.
30. When stepped over by a member of the household. 31. When you or a household member retrieves food or objects stolen by the dog.
32. Towards another (familiar) dog in your household (leave blank if no other dogs).
34. When approached while eating by another (familiar) household dog (leave blank if no other dogs).
35. When approached while playing with/chewing a favourite toy, bone, object, etc., by another (familiar) household dog (leave blank if no other dogs).
Section 3: Fear and anxiety (severity)
36. When approached directly by an unfamiliar adult while away from your home.
37. When approached directly by an unfamiliar child while away from your home.
38. In response to sudden or loud noises (e.g. vacuum cleaner, car backfire, road drills, objects being dropped, etc.).
39. When unfamiliar persons visit your home.
40. When an unfamiliar person tries to touch or pet the dog.
41. In heavy traffic
42. In response to strange or unfamiliar objects on or near the sidewalk (e.g. plastic trash bags, leaves, litter, flags flapping, etc.
43. When examined/treated by a veterinarian.
44. During thunderstorms, firework displays, or similar events.
45. When approached directly by an unfamiliar dog of the same or larger size.
46. When approached directly by an unfamiliar dog of a smaller size.
47. When first exposed to unfamiliar situations (e.g. first car trip, first time in elevator, first visit to veterinarian, etc.)
48. In response to wind or wind-blown objects.
49. When having nails clipped by a household member.
50. When groomed or bathed by a household member.
51. When having his/her feet towelled by a member of the household.
52. When unfamiliar dogs visit your home.
53. When barked, growled, or lunged at by an unfamiliar dog.
Section 4: Separation-related behaviour (frequency)
54. Shaking, shivering or trembling.
55. Excessive salivation.
56. Restlessness/agitation/pacing.
57. Whining.
58. Barking.
59. Howling.
60. Chewing/scratching at doors, floor, windows, curtains, etc.
61. Loss of appetite.
Section 5: Excitability (severity)
62. When you or other members of the household come home after a brief absence.
63. When playing with you or other members of your household.
64. When doorbell rings.
65. Just before being taken for a walk.
66. Just before being taken on a car trip.
67. When visitors arrive at your home.
Section 6: Attachment and Attention-seeking. (frequency)
68. Displays a strong attachment for one particular member of the household.
69. Tends to follow you (or other members of household) about the house, from room to room.
70. Tends to sit close to, or in contact with, you (or others) when you are sitting down.
71. Tends to nudge, nuzzle or paw you (or others) for attention when you are sitting down.
72. Becomes agitated (whines, jumps up, tries to intervene) when you (or others) show affection for another person.
73. Becomes agitated (whines, jumps up, tries to intervene) when you show affection for another dog or animal.
Section 7: Miscellaneous (frequency)
74. Chases or would chase cats given the opportunity.
75. Chases or would chase birds given the opportunity.
76. Chases or would chase squirrels, rabbits and other small animals given the opportunity.
77. Playful, puppyish, boisterous.
78. Active, energetic, always on the go.

Participants were invited to complete the C-BARQ online using Google Forms ^TM^ or in person through paper questionnaires distributed throughout the Greater Lisbon Metropolitan Area to various small animal hospitals, clinics and anti-rabies vaccination programs. Owners were instructed to complete the questionnaire as thoroughly as possible, however if they had no experience with the behaviour described, they were given the option to select “non-applicable” or “not observed”; these responses were treated as missing values in statistical analyses. Questionnaires were made available for a period of 8 months, resulting in 344 completed questionnaires.

### Statistical analysis

To assess the construct validity of the European Portuguese version of the C-BARQ, data obtained was subjected to principle components analysis using IBM SPSS Statistics version 20.0 (IBM Corp., Armonk, NY). To evaluate the reliability and to examine the internal consistency, the Cronbach’s alpha was used and interpreted according to DeVellis’ [30] criteria. To determine the number of interpretable factors that could be extracted through principal components analysis and varimax rotation, the Kaiser-Guttman eigenvalue method (eigenvalues greater than 1.0) and the Scree test were used. Loading values of 0.40 and greater were considered significant [31]. To study the internal validity of the C-BARQ, as relates to its construct validity, correlations between C-BARQ factors were calculated and item-factor correlations (point-biserial correlations) were examined to analyse the convergence of each item in the factor as well as its discrimination index (Nunnally and Bernstein, 1994). Correlations were analysed through the Pearson´s *r* coefficient. Missing values were treated as recommended by the original C-BARQ authors: if less than 25% of the items in a subscale were missing, the mean value of the subscale score was used throughout the data analysis (13).

## Results

### Population

The canine population under study was varied and is detailed in Table 2.

**Table 2:** Demographic characteristics of canine population in study (*N* = 345).

Age (years)	N (%)
<1	18(5)
1–5	132(38)
>5–10	96(28)
>10–15	80(23)
>15	19(6)
Sex	
Male	120(35)
Castrated Male	47(14)
Female	81(23)
Spayed Female	97(28)
Breed	
Specific breed cited	185(10)
Cross-breed	34(31)
Mutt	106(54)
No response	20(6)
Weight (kilograms)	
0–10	93(27)
11–25	146(42)
26–44	94(27)
>44	12(3)

### Factor analysis

Through analysis of the correlation matrix using the Kaiser-Meyer-Olkin measure of sampling adequacy, a value of 0.812 was obtained [32], and a significant Bartlettˋs test of sphericity (χ2 = 12071.958; df = 3003; p<0.001) confirmed that the sample size is adequate for analyses using principal components analysis [33,34].

The scree plot and eigenvalues suggested a 13-factor structure, which were extracted with item loadings presented in Table 3. This structure explained 58.42% of the total variance. Most of the items loaded onto the same factors as the original study [13], with the exception of two factors and two items (as shown in Table 3). In Duffy and Serpell´s [13] study Dog-directed Aggression (DDA) and Dog-directed Fear (DDF) had 4 items loading onto two different factors, whereas in the current study all 8 items loaded onto a single factor renamed Dog-directed Fear/Aggression [12]. Duffy and Serpell [13] loaded item 8 onto the factor “Trainability” (TR, factor 10) whereas in the current study the item loaded onto the factor “Energy” (EL). Item 43 in the Duffy and Serpell [13] study loaded onto the “Touch Sensitivity” (TS) factor, whereas in the current study the item loaded onto the “Non-social Fear” (NSF) factor.

**Table 3:** Results of factor analysis on the European Portuguese CBARQ.

Factors	α	eigenvalue	% variance	loadings
Factor 1 –Stranger directed aggression (SA)	0.90	6.33	8.12	
10. When approached directly by an unfamiliar adult while being walked/exercised on a leash				0.810
16. When unfamiliar persons approach you or another member of our family away from your home.				0.775
21. When an unfamiliar person tires to touch or pet the dog.				0.765
28. Toward unfamiliar persons visiting your home.				0.760
12. Toward unfamiliar persons approaching the dog while s/he is in your car (at the gas station for example).				0.693
15. When an unfamiliar person approaches you or another member of our family at home.				0.691
20. When strangers walk past your home while your dog is outside or in the yard.				0.685
18. When mailmen or other delivery workers approach your home.				0.633
22. When joggers, cyclists, rollerbladers or skateboarders pass your home while your dog is outside or in the yard.				0.611
11. When approached directly by and unfamiliar child while being walked/exercised on a leash.				0.568
Factor 2 –Dog-directed aggression/fear (DAF)*	0.86	3.98	5.11	
45. When approached directly by and unfamiliar dog of the same or larger size.				0.782
46. When approached directly by and unfamiliar dog of a smaller size.				0.777
53. When barked, growled, or lunged at by an unfamiliar dog.				0.698
52. When unfamiliar dogs visit your home.				0.663
23. When approached directly by an unfamiliar male dog while being walked/exercised on a leash.				0.623
24. When approached directly by and unfamiliar female dog while being walked/exercised on a leash.				0.571
26. Toward unfamiliar dogs visiting your home.				0.536
29. When barked, growled, or lunged at by another (unfamiliar) dog.				0.461
Factor 3 –Owner-directed aggression (ODA)	0.82	3.76	4.82	
19. When his/her food is taken away by a household member.				0.816
13. When toys, bones or other objects are taken away by a household member.				0.773
17. When approached directly by a household member while s/he (the dog) is eating.				0.771
31. When you or a household member retrieves food or objects stolen by the dogs.				0.674
9. When verbally corrected or punished (scolded, shouted at, etc.) by you or a household member.				0.489
25. When stared at directly by a member of the household.				0.452
14. When bathed or groomed by a household member.				0.434
30. When stepped over by a member of the household.				0.366
Factor 4 –Excitability (EX)	0.84	3.65	4.69	
65. Just before being taken for a walk.				0.789
66. Just before being taken on a car trip.				0.771
62. When you or other members of the household come home after a brief absence.				0.689
63. When playing with you or other members of your household.				0.667
67. When visitors arrive at your home.				0.614
64. When the doorbell rings.				0.535
Factor 5 –Stranger-directed fear (SDF)	0.90	3.44	4.40	
40. When an unfamiliar person tires to touch or pet the dog.				0.841
36. When approached directly by and unfamiliar adult while away from your home.				0.790
39. When unfamiliar persons visit your home.				0.785
37. When approached directly by an unfamiliar child while away from your home.				0.767
Factor 6 –Separation-related behaviour (SRB)	0.76	3.38	4.34	
57. Whinning.				0.699
59. Howling.				0.647
58. Barking.				0.633
54. Shaking, shivering or trembling.				0.623
56. Restlessness/agitation/pacing.				0.597
60. Chewing/scratching at doors, floors, windows, curtains, etc.				0.521
55. Excessive salivation.				0.477
61. Loss of appetite.				0.442
Factor 7 –Non-social fear (NSF)	0.78	3.26	4.17	
48. In response to wind or wind-blown objects.				0.705
38. In response to sudden or loud noises (e.g. vacuum cleaner, car backfire, road drills, objects being dropped, etc.).				0.641
44. During thunderstorms, firework displays, or similar events.				0.633
42. In response to strange or unfamiliar objects on or near the sidewalk (e.g. plastic trash bags, leaves, litter, flags flapping, etc.).				0.614
47. When first exposed to unfamiliar situations (e.g. first car trip, first time in elevator, first visit to veterinarian, etc.).				0.491
43. When examined/treated by a veterinarian. *				0.479
41. In heavy traffic.				0.412
Factor 8 –Dog rivalry/familiar dog aggression (DR)	0.87	3.24	4.15	
33. When approached at a favourite resting/sleeping place by another (familiar) household dog.				0.802
34. When approached while eating by another (familiar) household dog.				0.763
35. When approached while playing with/chewing a favorite toy, bone, object, etc., by another (familiar) household dog.				0.757
32. Towards another (familiar) dog in your household.				0.734
Factor 9 –Chasing (CH)	0.87	3.20	4.10	
76. Chases or would chase squirrels, rabbits and other small animals given the opportunity.				0.880
75. Chases or would chase birds give the opportunity.				0.844
74. Chases or would chase cats given the opportunity.				0.812
27. Towards casts, squirrels or other small animals entering your yard.				0.604
Factor 10 –Trainability (TR)	0.72	3.06	3.93	
1. When off the leash, returns immediately when called				0.607
3. Obeys the “stay” command immediately.				0.597
4. Seems to attend/listen closely to everything you say or do.				0.580
2.Obeys the “sit” command immediately.				0.579
7. Easily distracted by interesting sights, sounds or smells.				0.544
5. Slow to respond to correction or punishment; “thick-skinned”.				0.531
6. Slow to learn new tricks or tasks				0.516
Factor 11 –Attachment/attention-seeking behaviour (AAS)	0.75	2.88	3.69	
71. Tends to nudge, nuzzle or paw you (or others) for attention when you are sitting down.				0.661
70. Tends to sit close to, or in contact with, you (or others) when you are sitting down.				0.605
69. Tends to follow you (or other members of the household) about the house, from room to room.				0.601
68. Displays a strong attachment for one particular member of the household.				0.586
72. Becomes agitated (whines, jumps up, tries to intervene) when you (or others) show affection for another person.				0.538
73. Becomes agitated (whines, jumps up, tires to intervene) when you show affection for anther dog or animal.				0.506
Factor 12 –Energy level (EL)	0.81	2.75	3.53	
77. Playful, puppyish, boisterous.				0.806
78. Active, energetic, always on the go.				0.734
8. Will “fetch” or attempt to fetch sticks, balls, or objects. *				0.696
Factor 13 –Touch sensitivity (TS)	0.73	2.64	3.38	
51. When having his/her feet towelled by a member of the household.				0.745
50. When groomed or bathed by a household member.				0.724
49. When having nails clipped by a household member.				0.682

*denotes differences between factors/items in the current study and in the orginal [13]

In all cases, items loading values were above 0.412, with the exception of item 30 (loading 0.366). Despite this lower value, the item represented at least 9% of the variance accounted for in the factor [35] and as such was maintained.

### Internal consistency and internal validity

The internal consistency of extracted factors was analysed using Cronbach’s alpha, with values above 0.70 considered to have adequate reliability. The Cronbach’s alpha values ranged from 0.902 and 0.721 (Table 3), showing excellent to respectable consistency [30].

Significant correlations (p<0.01, p<0.05) were found between the 13 factors within the C-BARQ; the coefficients, which varied between 0.454 and 0.108, denoting mostly moderate or weak correlations [36]. However, some negative coefficients (in a few weak associations) and null associations were also detected. Item-factor correlations can be found in Table 4.

**Table 4:** Item-factor correlation summary.

Factor	Coefficient Variation	M
SA	0.74–0.52	0.65*
DAF	0.68–0.52	0.60*
ODA	0.71–0.42	0.56†
EX	0.61–0.53	0.63*
SDF	0.83–0.75	0.79*
SRB	0.55–0.37	0.47†
NSF	0.61–0.37	0.51†
DR	0.76–0.63	0.72*
CH	0.81–0.57	0.73*
TR	0.49–0.36	0.43‡
AAS	0.55–0.41	0.49†
EL	0.74–0.56	0.67*
TS	0.67–0.52	0.58*

M = mean

*strong

†strong to moderate

‡ moderate [36] Note: SA = Stranger-Directed Aggression, DAF = Dog-Directed Aggression/Fear, ODA = Owner-Directed Aggression, Ex = Excitability, SDF = Stranger-Directed Fear, SRB = Separation-Related Behavior, NSF = Nonsocial Fear, DR = Dog Rivalry, CH = Chasing, TR = Trainability, AAS = Attachment/Attention-Seeking Behavior, EL = Energy Level, TS = Touch Sensitivity.

## Discussion

This paper set out to study the psychometric properties of the European Portuguese version of the C-BARQ to establish its validity for use in a European Portuguese context. The obtained results for this instrument suggest good validity and reliability indices, with a robust 13-item factor structure accounting for 58.42% of the total variance of the results. These findings reveal the important psychometric qualities of the instrument and highlight specific differences found in the current population compared to others studied.

The European Portuguese version of the C-BARQ very closely followed the structure of the original [13], with the extraction of almost all of the same subscales. The exception was the two subscales, DDA and DDF, each with 4 items loading strongly onto one factor that we renamed Dog Associated Fear/Aggression (Table 4). Although this result was similar to the results obtained by Svartberg [11], it contrasts clearly with studies carried out in other countries [19–21]. Portugal has only recently started to see the dog as a family member, and many dogs are still kept in yards. The importance of socializing dogs [37] is not widely acknowledged by Portuguese owners and, as a result, some dogs may show inappropriate behaviour when meeting an unfamiliar animal making the line between aggression and fear difficult to draw. This inexperience with dog behaviour could account for the grouping of DAF and DAA into a single factor.

When considering individual items on the European Portuguese C-BARQ, each loaded strongly on its expected subscale, except for two: items 8 and 43. The former, “will fetch or attempt to fetch sticks, balls, or objects” loaded onto the subscale EL instead of the original TR [13] as it did in a recent Mexican study [24]. In Portugal, dog training classes have only recently started to be regularly offered and, as in other countries, few owners attend [38]. It is possible that fetching is not considered to be an act of training but of playing. Dogs scoring high on the EL factor may tend towards a more extroverted personality [39] and may readily display fetch-like behaviours, but may not have been receptive to basic obedience commands such as “sit” and “stay”, items included in the TR factor. Item 8 is also the only TR subscale item that can be demonstrated by the dog when alone, making it more likely to be displayed by extroverted, high energy dogs.

The only other item that differed from the English C-BARQ was “when examined/treated by a veterinarian” (item 43), which loaded onto the subscale NSF instead of the original TS subscale [13]. As previously suggested [19], the reaction of a dog when examined by a veterinarian may not be an accurate measurement of touch sensitivity, but rather of fear, as the dog could be reacting as a result of a previous negative experience with veterinarians. It is the only item in the TS subscale that involves a potentially unfamiliar person, and the dog could be effectively reacting to fear of a novel person. This may be even more true in Portugal, where visits to veterinarians have traditionally been exclusively for obligatory rabies vaccinations instead of regular health care checks during the dog’s entire lifetime.

While great care was taken to try and obtain the most representative dog owner population possible by distributing the questionnaire in every parish in the Greater Lisbon Metropolitan Area, spanning a wide variety of socioeconomic classes, it must be noted that the experimental design required that owners volunteer to participate. As stated by various authors [19,38,40] these owners may be naturally more connected with their dogs, making them more observant than the general population. Although this effect can never be completely accounted for, the fact that almost identical factor structures where extracted from data in different countries [19–21,24,41] gives weight to the notion that the questionnaire does measure universal dog behaviours that are evident to most owners, regardless of individual characteristics, such as culture or attachment level.

## Conclusion

The C-BARQ has been shown to be an effective instrument, both valid and reliable, that can be used cross culturally. Small differences that may arise between countries can be identified by validating new translated versions of the questionnaire before they are widely used [19]. This study has demonstrated that the European Portuguese version of the C-BARQ can confidently be used to help characterize the behaviour of the Portuguese dog population and, as such, direct any future public education endeavours. This is borne out by the excellent psychometric properties demonstrated both in terms of reliability and validity. The instrument is adequate for use in animal shelters to better match dogs with potential new owners and in clinical settings to identify behaviour problems in veterinary patients before they become unmanageable. The European Portuguese C-BARQ could be of vital importance to help resolve behavioural problems in owned dogs before they become so serious as to lead to abandonment or euthanasia, diminishing the pressure on municipal kennels and greatly improving canine welfare in Portugal.

## Supporting information

S1 Table Original responses to European Portuguese C-Barq. (DOCX)
