# Supplementary material for: Evaluation of the factor structure of the Canine Behavioural Assessment and Research Questionnaire (C-BARQ) in European Portuguese
Source: PLoS One. 2018 Dec 27;13(12):e0209852. doi: 10.1371/journal.pone.0209852 (PMC6307869; doi:10.1371/journal.pone.0209852)
Supplement: S1 Table — (DOCX) [file pone.0209852.s001.docx]

Table 1: C-BARQ descriptive statistics.

| Factors | M | SD | Min. | Max. | Skewness | Kurtosis |
| --- | --- | --- | --- | --- | --- | --- |
| SA (10 items) | 5.57 | 6.42 | 0 | 32 | 1.58 | 2.49 |
| DAF (8 items) | 7.95 | 6.17 | 0 | 32 | 1.01 | 0.99 |
| ODA (8 items) | 1.30 | 2.89 | 0 | 19 | 3.47 | 13.70 |
| Ex (6 items) | 14.08 | 5.26 | 0 | 24 | -0.18 | -0.56 |
| SDF (4 items) | 2.12 | 3.09 | 0 | 15 | 1.86 | 3.26 |
| SRB (8 items) | 4.88 | 4.63 | 0 | 26 | 1.21 | 1.56 |
| NSF (7 items) | 6.31 | 4.86 | 0 | 23 | 0.83 | 0.09 |
| DR (4 items) | 1.79 | 2.79 | 0 | 16 | 2.51 | 7.50 |
| Ch (4 items) | 7.27 | 4.79 | 0 | 16 | 0.20 | -1.02 |
| TR (7 items) | 18.10 | 4.30 | 7 | 28 | -0.19 | -0.26 |
| AAS (6 items) | 14.16 | 4.37 | 1 | 24 | -0.05 | -0.17 |
| EL (3 items) | 8.10 | 3.08 | 0 | 12 | -0.59 | -0.48 |
| TS (3 items) | 1.95 | 2.25 | 0 | 12 | 1.52 | 2.65 |

Note: SA=Stranger-Directed Aggression, DAF=Dog-Directed Aggression/Fear, ODA=Owner-Directed Aggression, Ex=Excitability, SDF=Stranger-Directed Fear, SRB=Separation-Related Behavior, NSF=Nonsocial Fear, DR=Dog Rivalry, CH=Chasing, TR=Trainability, AAS=Attachment/Attention-Seeking Behavior, EL=Energy Level, TS=Touch Sensitivity. M (mean), SD (standard-deviation).
